# Clinical usefulness of inside stents in anastomotic biliary strictures after liver transplantation

**DOI:** 10.1002/deo2.305

**Published:** 2023-10-27

**Authors:** Naotaka Kugiyama, Shunpei Hashigo, Katsuya Nagaoka, Takehisa Watanabe, Shinya Ushijima, Yukiko Uramoto, Motohiro Yoshinari, Jun Morinaga, Ryosuke Gushima, Masakuni Tateyama, Motohiko Tanaka, Hideaki Naoe, Yasuhiko Sugawara, Taizo Hibi, Yasuhito Tanaka

**Affiliations:** ^1^ Department of Gastroenterology and Hepatology Kumamoto University Hospital Kumamoto Japan; ^2^ Department of Clinical Investigation Kumamoto University Hospital Kumamoto Japan; ^3^ Department of Public Health and Welfare Kumamoto Japan; ^4^ Department of Pediatric Surgery and Transplantation Kumamoto University Hospital Kumamoto Japan

**Keywords:** biliary stricture, endoscopic biliary stenting, inside stent, liver transplantation, period of stent patency

## Abstract

**Background:**

Endoscopic biliary stenting is a standard treatment for biliary strictures after liver transplantation. Plastic stents are often replaced before stent dysfunction to prevent the development of cholangitis and jaundice. Therefore, the precise duration of stent patency is unclear.

**Methods:**

We compared retrospectively the stent patency period and stent dysfunction rate between inside stents (IS) and conventional plastic stents (PS) in 48 patients with post‐transplant strictures, distinguishing endoscopic biliary stenting with and without stent dysfunction at stent replacement.

**Results:**

In observations focused on the first treatment, the median patency periods were 369 days for IS (*n* = 18) and 154 days for PS (*n* = 30; *p* = 0.01), significantly longer for IS. The 1‐year cholangitis incidence rate was lower for IS (20% vs. 43%, *p* = 0.04). Additionally, no stent dislocation was observed for IS, but this occurred for 33.3% of PS (*p* = 0.004). Comparing all endoscopic biliary stenting, including second and subsequent procedures, IS again had a longer patency period than PS (356 days, *n* = 89, vs. 196 days, *n* = 127, *p* = 0.009).

**Conclusions:**

IS had a significantly longer patency period than PS, suggesting that IS replacement could be reduced to once per year for patients who prefer less frequent stent replacement.

## INTRODUCTION

Anastomotic biliary stricture is the most serious complication of liver transplantation. Living donor liver transplantation (LDLT) is more common than deceased donor LT (DDLT) in Japan.^1^ Duct‐to‐duct reconstruction is commonly performed, and biliary strictures occur in 12%–30% of patients after LDLT^2^, ^3^, ^4^, ^5^, ^6^ and in 9%–12% of patients after DDLT.^7^, ^8^ For biliary strictures, endoscopic retrograde cholangiography (ERC)‐related procedures, such as stent placement and balloon dilation, have been widely adopted as standard treatment.^9^ Patients who do not achieve complete improvement with these treatments require stent placement and regular replacement to keep their bile ducts patent.

Apart from improving occluding biliary strictures, plastic stents are often replaced strategically for benign strictures, even without occlusion, as a preemptive measure to avoid problems of obstruction in advance. The precise period of stent patency is not obvious in such cases. Previous reports^10^, ^11^ evaluating the stent patency period after transplantation did not distinguish between regular replacement without stent occlusion and emergency replacement after occlusion; this makes it difficult to accurately assess the duration of stent patency. Therefore, we focused on evaluating the period of stent patency more accurately by distinguishing between ERC with and without stent dysfunction. For patients with no improvement in stricture and requiring regular stent replacement, there is a lack of epidemiological knowledge regarding which stent type has the longer patency period and when it is appropriate to replace.

Conventional plastic stents (PS), that are placed across the papilla with the distal end exposed to the duodenum, carry the risk of occlusion because of reflux of duodenal contents.^12^, ^13^ On the other hand, inside stents (IS) are expected to reduce the risk of stent occlusion, without exposure to the duodenum contents, because the distal end is placed in the bile duct.^10^, ^14^, ^15^ We assess retrospectively the precise periods of stent patency by dealing with replacement without stent dysfunction as a censoring event, using the log‐rank test, and compare the different outcomes for IS and PS.

## METHODS

### Study cohort

From January 2009 to April 2021, we analyzed 48 patients with successful endoscopic biliary stenting (EBS), among 51 patients who underwent ERC to treat biliary strictures after liver transplantation with duct‐to‐duct biliary reconstruction in Kumamoto University Hospital. A total of 216 EBS were accessed retrospectively. Biliary strictures were diagnosed by ERC. Some patients and EBS were excluded. Exclusion criteria were: (1) Cases with non‐anastomotic strictures; (2) Cases with IS and PS placement at the same time; (3) Cases in which an endoscopic nasobiliary drainage tube was placed; (4) Cases in which a self‐expanding metallic stent (SEMS) was placed.PS were used mainly from 2009 to 2015 and IS placement was started from 2016, when it became available in our hospital, with the aim of reducing the incidence of retrograde cholangitis. The study was approved by the Institutional Review Board of Kumamoto University (acceptance number 2236).

### Endoscopic treatment and follow‐up

**FIGURE 1:**
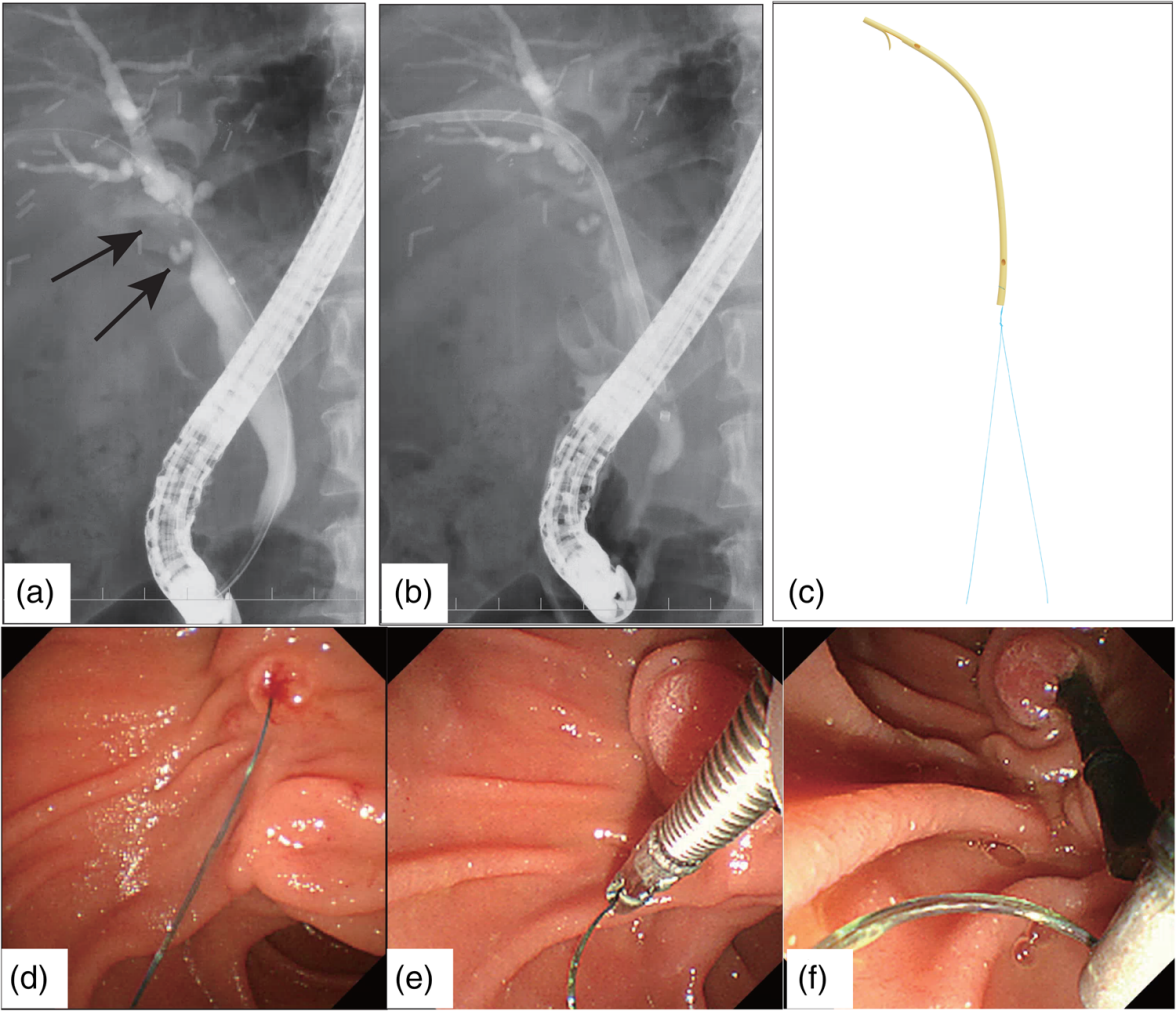
Endoscopic biliary stenting for biliary stricture after liver transplantation. (a) Endoscopic retrograde cholangiography (ERC) shows the biliary stricture (arrow) after liver transplantation with duct‐to‐duct biliary reconstruction. (b) The inside stent is placed across the strictures and the lower end is in the common bile duct. (c) The inside stent (Gadelius Medical), the suture thread is tied to the distal end of the stent. (d) The stent is placed above the papilla and the lower end of the stent is not exposed in the duodenum. (e, f) The suture at the distal end was pulled and removed with forceps when necessary.

After confirming the stricture by ERC, 4–8 mm balloon dilation was performed and, the stent sizes, numbers, and lengths were determined and then placed, depending on the cholangiography. PS (Straight type, Flexima; Boston Scientific Japan) were placed across the sphincter of Oddi, with their distal ends exposed into the duodenum. IS (Straight type, Through&Pass; Gadelius Medical) were placed above the sphincter of Oddi (Figure 1). To reduce the risk of cholangitis due to reflux of duodenal contents, endoscopic sphincterotomy (EST) was not performed for either group, in principle. After EBS, the patients were followed up with blood tests every 1–2 months and ERC was usually scheduled every 3–12 months. Other than strategic stent replacement, ERC was performed in the case of any signs of stent occlusion, acute cholangitis, or stent dislocation. At the next ERC after EBS, if the stricture remained, IS or PS were placed after balloon dilation. If the stricture had improved, the duct was left stent‐free. ERC complications were diagnosed by blood tests and physical examination the day after ERC.

### Primary outcome and evaluation procedure

For the first EBS to the biliary stricture, the stent patency period and the 1‐year stent dysfunction rate were compared between the patients given IS and PS. Next, we compared the stent patency period and the 1‐year stent dysfunction rate for each endoscopic procedure, between the IS and PS groups, not limited to the first treatment but including all individual procedures.

The stent patency period was defined as the time interval from the day of stent placement to the next ERC and analyzed using the Kaplan‐Meier method, based on the TOKYO Criteria 2014 for evaluating stent patency in malignant biliary strictures. The strategic replacements without stent dysfunction were censored at the time of analysis. Stent dysfunction was defined as stent occlusion, stent dislocation, or acute cholangitis. Stent occlusion was defined as the elevation of hepatobiliary enzymes above normal, in the absence of fever. Acute cholangitis was defined as the elevation of hepatobiliary enzymes above normal with fever (higher than 37°C) or the use of antibiotics. Stent dislocation was defined as the movement of the stent to a site other than the biliary stricture. The 1‐year stent occlusion rate was defined as the proportion of stent occlusions at 1 year after stent placement, analyzed using the Kaplan‐Meier method. In the analysis of the 1‐year stent occlusion rate, all ERCs except “stent occlusion” were censored according to the Kaplan‐Meier method. Specifically, “Strategic replacements (no event occurred)”, “ERC with acute cholangitis”, and “ERC with stent dislocation” were censored. Similarly, the 1‐year acute cholangitis rate and 1‐year stent dislocation rate were defined as the proportion of acute cholangitis and stent dislocation at 1 year after stent placement.

### Statistical analysis

Statistical analyses were performed using SPSS, version 25.0 (SPSS, Inc.). The period of stent patency and 1‐year stent dysfunction rate were analyzed using the Kaplan‐Meier method. The log‐rank test was used for comparisons between the IS and the PS groups. Differences of *p* < 0.05 were considered statistically significant.

## RESULTS

### Characteristics of the patients

We analyzed 48 patients who underwent successful EBS. The characteristics of the patients, transplantation procedure, and stricture improvement rates are shown in Table 1. The most common indication for liver transplantation was liver cirrhosis (52.1%). Biliary strictures occurred in five patients after DDLT and 43 after LDLT. The median interval from transplantation to the first ERC was 234 days (range, 83–5816 days). The median follow‐up period after the first ERC was 1059 days (range, 34–3647) and the median number of EBS was 4 (range, 1–19). The stricture improved in 21 patients (44%) on repeat EBS, the median number of EBS was 3 (range, 2–8); however, stricture recurrence was observed in eight patients (38%). Of the 21 patients with stricture improvement, there were 19 who had received LDLT, two who had received DDLT, five patients received IS, and 16 received PS in the first EBS. Moreover, strictures improved in only five patients within around a year. There were no significant differences between the characteristics of patients who received IS and those with PS in the first ERC.

**TABLE 1:** Characteristics of the patients.

	Total	IS^*^	PS^*^	
	*n* = 48	*n* = 18	*n* = 30	*p‐*value
Age, years (range)	56 (10–75)	54 (10–75)	56 (15–66)	0.25
Sex, male/female, *n*	30/18	10/8	20/10	0.44
Indication for liver transplantation, *n* (%)				0.57
Liver cirrhosis	25 (52.1%)	7 (38.9%)	18 (60.0%)	
Associated hepatocellular carcinoma	10 (20.8%)	5 (27.8%)	5 (16.7%)	
Metabolic liver disease	5 (10.4%)	2 (11.1%)	3 (10.0%)	
Fulminant hepatitis	3 (6.3%)	1 (5.6%)	2 (6.7%)	
Others	5 (10.4%)	3 (16.7%)	2 (6.7%)	
LDLT/DDLT, *n*	43/5	15/3	28/2	0.27
Donated liver, *n* right/left/posterior/whole liver	28/10/5/5	9/5/1/3	19/5/4/2	0.43
Number of anastomotic bile ducts, *n* 1/2/3	28/19/1	10/8/0	18/11/1	0.66
Interval from transplantation to ERC, days (range)	234 (83–5816)	189 (83–5816)	261 (87–1651)	0.53
Number of stricture improvements, *n* (%)	21 (44%)	5 (28%)	16 (53%)	0.08
Number of stricture recurrences, *n* (%)	8 (38%)	2 (40%)	6 (38%)	0.92

Abbreviations: DDLT, deceased donor liver transplantation; ERC, endoscopic retrograde cholangiography; IS, inside stent; LDLT, living donor liver transplantation; PS, plastic stent.

* The stent placed in the first ERC for biliary stricture after liver transplantation.

### The IS group experienced long‐term stent patency after the first ERC

**FIGURE 2:**
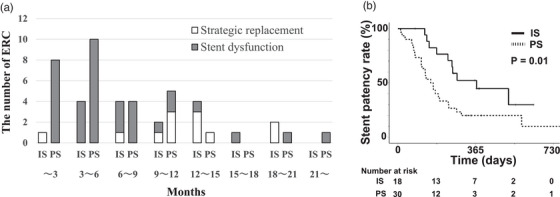
The numbers of endoscopic retrograde cholangiographies (ERCs) for the two groups, at 3‐month intervals, and the Kaplan–Meier curves showing the stent patency rates. (a) The numbers of ERCs for the two groups at three‐monthly intervals. Three patients in each group underwent strategic replacement without stent dysfunction within a year (*p* = 0.66). (b) After the first endoscopic biliary stenting (EBS), the Kaplan–Meier curves show the stent patency rates of the inside stents (IS) and plastic stents (PS) groups. The period of stent patency was significantly longer for the IS group, with the median period of stent patency being 369 days.

Considering the first treatment after transplantation, 18 patients received IS (7–8.5 Fr) and 30 received PS (5–8.5 Fr). The number of stents, stent size, and ERC complications are shown in Table 2a; there was no difference in ERC complications. There were 15 ERCs in the IS group up to 15 months after stent placement and 28 ERCs in the PS group, and the ERC rate within 15 months for all ERCs was 83.3% in the IS group and 93.3% in the PS group (*p* = 0.35). Three patients in each group underwent strategic replacement without stent dysfunction within a year (*p* = 0.66). The numbers of ERC at 3‐month intervals are shown in Figure 2a.

**TABLE 2a:** Treatments and complications in the first endoscopic retrograde cholangiography.

	Total	IS	PS	
	*n* = 48	*n* = 18	*n* = 30	*p‐*value
Number of stents, 1/2, *n*	39/9	13/5	26/4	
Stent size, 5 Fr/7 Fr/8.5 Fr, *n*	4/34/10	0/12/6	4/22/4	
Post‐ERC complications, *n* (%)	4 (8.3%)	1 (5.6%)	3 (10%)	1.00
Pancreatitis	2 (4.2%)	0 (0%)	2 (6.7%)	0.52
Hyperamylasemia	2 (4.2%)	1 (5.6%)	1 (3.3%)	1.00
Acute cholangitis	0 (0%)	0 (0%)	0 (0%)	NA
Elevation of biliary enzymes	0 (0%)	0 (0%)	0 (0%)	NA

Abbreviations: ERC, endoscopic retrograde cholangiography; IS, Inside stent; PS, plastic stent.

The median period of stent patency was 369 days for the IS group and 154 days for the PS group (*p* = 0.01, hazard ratio: 0.40), significantly longer for the IS group (Figure 2b). The stent patency periods and 1‐year stent dysfunction rates are shown in Table 2b. From subgroup analysis under the same conditions, the median period of stent patency with a 7Fr single stent was 254 days for the IS group (*n* = 7) and 154 days for the PS group (*n* = 18; *p* = 0.222).

**TABLE 2b:** The stent patency periods and 1‐year stent dysfunction rates for the first endoscopic retrograde cholangiography.

	Total	IS	PS	
	*n* = 48	*n* = 18	*n* = 30	*p‐*value
Median period of stent patency, days	196	369	154	0.01
Stent dysfunction factor				
Cases of stent occlusion within a year, *n* (%)		8 (44%)	14 (47%)	
One‐year stent occlusion rate, %		48%	65%	0.28
Cases of acute cholangitis within a year, *n* (%)		2 (11%)	9 (30%)	
One‐year acute cholangitis rate, %		20%	43%	0.04
Cases of stent dislocation within a year, *n* (%)		0 (0%)	10 (33%)	
One‐year stent dislocation rate, %		0%	43%	0.004

Abbreviations: IS, inside stent; PS, plastic stent.

The median periods of stent patency and 1‐year stent dysfunction rates were analyzed using the Kaplan‐Meier method (the log‐rank test).

**FIGURE 3:**
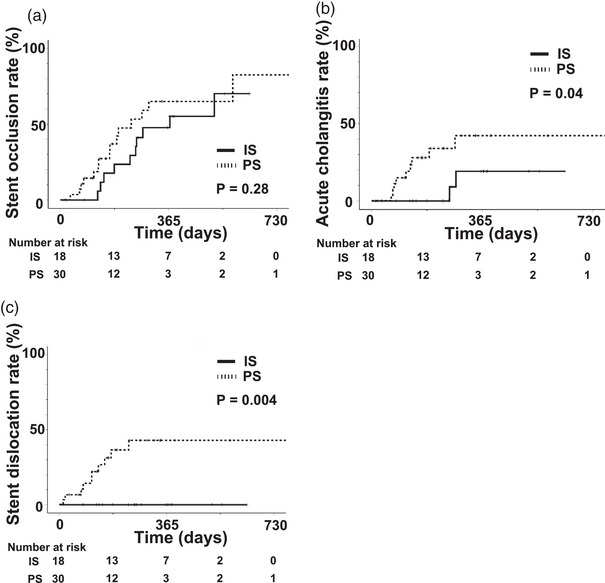
Comparison of stent occlusion rates, acute cholangitis rates, and stent dislocation rates between the inside stents (IS) and plastic stents (PS) groups after the first endoscopic biliary stenting (EBS). (a) The stent occlusion rates did not differ between the IS and PS groups. (b) The acute cholangitis rate was significantly lower in the IS group. (c) Stent dislocation occurred frequently in the PS group but never in the IS group.

Stent occlusion was observed within a year in eight patients (44%) in the IS group and 14 (47%) in the PS group, and the one‐year stent occlusion rates analyzed by the Kaplan‐Meier method were 48% in the IS group and 65% in the PS group (*p* = 0.28, Figure 3a). Acute cholangitis was observed within a year in two patients (11%) in the IS group and nine (30%) in the PS group, and the 1‐year acute cholangitis incidence rate was significantly lower in the IS group than the PS group (20% vs. 43%, *p* = 0.04, Figure 3b). In addition, no stent dislocation was observed in the IS group, but this did occur within a year in 10 patients (33.3%) in the PS group. The 1‐year stent dislocation rate was significantly lower in the IS group than in the PS group (0% vs. 43%, *p* = 0.004, Figure 3c).

### Analysis of all ERCs also supported longer stent patency with IS

**FIGURE 4:**
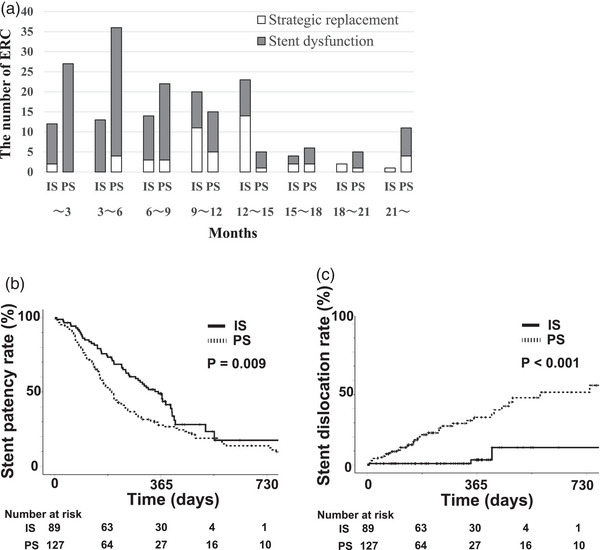
For the total endoscopic biliary stenting (EBS), the number of endoscopic retrograde cholangiographies (ERCs) at 3‐month intervals and comparison of stent patency rates and stent dislocation rates between the inside stents (IS) and plastic stents (PS) groups. (a) The numbers of ERCs for the two groups at three monthly intervals. The number of strategic replacements within a year, without stent dysfunction, was 16 (18.0%) for the IS group and 12 (9.4%) for the PS group (*p* = 0.098). (b) Similar to the first EBS, the period of stent patency was significantly longer for the IS group. (c) The stent dislocation rate was significantly lower for the IS group.

We carried out 89 treatments with IS (7–8.5 Fr) and 127 treatments with PS (5–10 Fr), as shown in Table 3a. The ERC rates within 15 months of stent placement for all ERCs were 92.1% in the IS group and 82.7% in the PS group (*p* = 0.07). The number of strategic replacements without stent dysfunction within a year was 16 (18.0%) in the IS group and 12 (9.4%) in the PS group (*p* = 0.098). The numbers of ERC at 3‐month intervals are shown in Figure 4a.

**TABLE 3a:** Treatments and complications in all endoscopic biliary stenting.

	Total	IS	PS	
	*n* = 216	*n* = 89	*n* = 127	*p‐*value
Number of stents, 1/2, *n*	161/55	50/39	111/16	
Stent size, 5Fr/7Fr/8.5Fr/10Fr, *n*	5/90/98/23	0/30/59/0	5/60/39/23	
Post‐ERC complications, *n* (%)	28 (13%)	17 (19.1%)	11 (8.7%)	0.025
Pancreatitis	5 (2.3%)	3 (3.4%)	2 (1.6%)	0.41
Hyperamylasemia	11 (5.1%)	3 (3.4%)	8 (6.3%)	0.53
Acute cholangitis	7 (3.2%)	7 (7.7%)	0 (0%)	0.002
Elevation of biliary enzymes	5 (2.3%)	4 (4.5%)	1 (0.8%)	0.16

Abbreviations: ERC, endoscopic retrograde cholangiography; IS, Inside stent; PS, plastic stent.

Similar to the comparison of patients after the first ERC, the median period of stent patency was 356 days in the IS group, significantly longer than the PS group (196 days; *p* = 0.009, hazard ratio: 0.64; Figure 4b). From subgroup analysis, the median periods of stent patency with a 7Fr single stent were 287 days (IS group, *n* = 19) and 188 days (PS group, *n* = 51), (*p* = 0.084), the IS group also tended to be better. The stent patency periods and 1‐year stent dysfunction rates are shown in Table 3b.

**TABLE 3b:** The stent patency periods and 1‐year stent dysfunction rates in all endoscopic biliary stenting (EBS).

	Total	IS	PS	
	*n* = 216	*n* = 89	*n* = 127	*p‐*value
Median period of stent patency, days	239	356	196	0.009
Stent dysfunction factor				
Cases of stent occlusion within a year, *n* (%)		41 (46%)	58 (46%)	
One‐year stent occlusion rate, %		49%	58%	0.66
Cases of acute cholangitis within a year, *n* (%)		12 (13%)	21 (17%)	
One‐year acute cholangitis rate, %		17%	21%	0.29
Cases of stent dislocation within a year, *n* (%)		2 (2%)	30 (24%)	
One‐year stent dislocation rate		4%	34%	< 0.001

Abbreviations: IS, inside stent; PS, plastic stent.

The median periods of stent patency and 1‐year stent dysfunction rates were analyzed using the Kaplan‐Meier method (the log‐rank test).

Stent occlusion was observed within a year in 41 cases (46%) in the IS group and 58 cases (46%) in the PS group, and the 1‐year stent occlusion rates analyzed by the Kaplan‐Meier method were 49% in the IS group and 58% in the PS group (*p* = 0.66). Acute cholangitis was observed within a year in 12 cases (13%) in the IS group and 21 cases (17%) in the PS group, and the one‐year acute cholangitis incidence rates were 17% in the IS group and 21% in the PS group (*p* = 0.29). Stent dislocation was observed within a year in two cases (2%) in the IS group and 30 cases (24%) in the PS group, and the 1‐year stent dislocation rate was significantly lower in the IS group than in the PS group (4% vs. 34%, *p* < 0.001, Figure 4c). A comparison of post‐ERC complications shows a 19.1% complication rate in the IS group and 8.7% in the PS group. The rate of acute cholangitis was significantly higher in the IS group (Table 3a). However, no cases of severe cholangitis were observed, and all cases that showed elevated biliary enzymes recovered to normal levels within a few days.

## DISCUSSION

In this study, we assessed the period of stent patency more accurately by distinguishing between stent replacement with and without stent dysfunction. The median stent patency of IS was about 1 year, and IS has a longer patency period and a lower frequency of stent dysfunction than PS. These results make a novel contribution to the knowledge base to select IS or PS for patients with anastomotic stricture after liver transplantation.

The resolution rate of biliary strictures after transplantation has been reported to vary widely, from 20% to 100%, and the rate of recurrence of stricture is reportedly 10%–30%, during a median follow‐up period of 9.5–70 months.^16^, ^17^, ^18^, ^19^, ^20^ Moreover, some reports indicate that multiple stenting or SEMS are also useful for improving strictures, by providing greater dilation.^21^, ^22^ In this study, the stricture resolution rate was not high (44%), this is because large‐diameter stents and multiple stenting were used less frequently than in other studies. When the stricture does not improve after repeated endoscopic procedures, regular stent replacement should be continued to keep the bile duct patent. Cholangitis due to stent dysfunction should be avoided for patients who are immunosuppressed after transplantation. On the other hand, frequent ERCs possibly reduce the patient's quality of life. Stent (IS or PS) replacements are generally conducted every three months to improve the stricture and prevent stent occlusion^23^; however, for patients with no improvement of the stricture and who need semi‐permanent stent replacement, replacing the stent every three months is potentially burdensome.

According to some reports, the period of IS patency for anastomotic stricture after LDLT is around 161–222 days, with EBS scheduled every 6–12 months.^10^, ^11^, ^23^ In our study, the median IS patency period was longer (356 days). This is partly because past studies did not distinguish between strategic replacement without stent dysfunction and emergency replacement due to dysfunction when analyzing the Kaplan‐Meier method. IS occlusion in biliary stricture after LDLT was reported to occur in 16%–30% at 140–175 days,^10^, ^11^ and our observation of the 1‐year stent occlusion rate in the IS group was 49%. However, the 1‐year acute cholangitis rate in the IS group was not so high (17%) and nearly 40% of the IS group were exchanged without stent dysfunction. Hence, the median stent patency of IS was 356 days, and the incidence of acute cholangitis was 17% in 1 year, suggesting that IS replacement could be reduced to once per year for patients who prefer less frequent stent replacement. Note that ERC should be scheduled more frequently for patients whose priority is to avoid cholangitis.

In this study, the stent size and number varied between the two groups, because these were determined based on the degree of stricture. The subgroup analysis, limited to the standard stent diameter, 7Fr single stent, showed the IS group also tended to be better among the first and all EBS. These results also suggested that IS had a longer patency period than PS, independent of stent size.

We observed many stent dislocations in the PS group (Figures 3c and 4c); this is one of the reasons the patency period of the PS was so short. The plastic stent dislocation rate for benign stricture is reportedly 13.7%, which was higher than for malignant stricture.^24^ Biliary stricture may persist, even after stent dislocation, and the reasons for stent dislocation potentially include duodenal peristalsis, inappropriate stent length, and the number of stents. Although a pigtail stent may reduce the risk of dislocation in the PS group, the intrahepatic bile ducts often become narrow after LDLT, making it difficult to place this type of stent.

Acute cholangitis after EBS was predominant in the IS group. This is because EST had not been performed and the lower end of the stent was also located in the common bile duct, suggesting that temporary papilledema due to ERC potentially blocked the outflow of bile into the duodenum. However, no severe cholangitis was observed, and the biliary enzymes improved within a few days. We propose that IS should be selected if long‐term stent patency is required, even considering the possibility of biliary enzyme elevation.

There are some limitations to this study. First, it is a retrospective single‐center study with a small number of patients, and IS and PS were used during different periods. Although the statistical power to evaluate the effect of IS stent was sufficient in the current study (power > 0.8), further analysis with a large number of cases is necessary for greater accuracy. Secondly, the stent size and number varied between the two groups. This study also does not adequately assess confounding factors that may contribute to stent patency, such as transplantation procedure and patient background. A prospective RCT in multiple centers is required to select an appropriate stent, including type, size, and number.

In conclusion, IS in biliary stricture after liver transplantation provides longer periods of stent patency than PS, and the 1‐year acute cholangitis rate was 17%. Hence, IS should be selected for patients who require long‐term stent patency, and it is suggested that IS replacement could be reduced to once per year for patients who prefer less frequent stent replacement. However, patients whose priority is to avoid cholangitis should continue to undergo ERC for a short period of time. This study provides new evidence in the management of post‐transplant biliary stricture and may contribute to improving the quality of life of patients.

## CONFLICT OF INTEREST STATEMENT

None.
